# The Novel Crohn's Disease Marker Anti-GP2 Antibody Is Associated with Ileocolonic Location of Disease

**DOI:** 10.1155/2013/683824

**Published:** 2013-05-15

**Authors:** Valentina Somma, Hani Ababneh, Ahmad Ababneh, Simona Gatti, Vittorio Romagnoli, Emanuele Bendia, Karsten Conrad, Dimitrios P. Bogdanos, Dirk Roggenbuck, Gino Ciarrocchi

**Affiliations:** ^1^R/D, Medipan GmbH, 15827 Dahlewitz/Berlin, Germany; ^2^Immunology Department, King Hussein Medical Center, Amman 11855, Jordan; ^3^University of Jordan, Amman 11942, Jordan; ^4^Department of Pediatrics, Polytechnic University of Marche, 60123 Ancona, Italy; ^5^Department of Gastroenterology, “Ospedali Riuniti” University Hospital, 60020 Ancona, Italy; ^6^Institute of Immunology, Technical University, 01307 Dresden, Germany; ^7^Division of Transplantation Immunology and Mucosal Biology, Institute of Liver Studies, King's College London School of Medicine at King's College Hospital, Denmark Hill Campus, London SE5 9RJ, UK; ^8^Faculty of Natural Sciences, Lausitz University of Applied Sciences, 01968 Senftenberg, Germany; ^9^Central Analytical Laboratory, “Ospedali Riuniti” University Hospital, 60020 Ancona, Italy

## Abstract

Crohn's disease (CD) is an inflammatory bowel disease (IBD) that can affect the whole gastrointestinal tract. The ileocolonic variant of CD, an inflammation of both the ileum and the large intestine, accounts for up to 50% of the cases with CD, whereas Crohn's ileitis affecting the ileum is diagnosed in about 30%. Crohn's colitis, which is confined to the large intestine and accounts for the remaining 20%, is difficult to distinguish from the large bowel inflammation seen in patients with ulcerative colitis (UC). The pathogenesis of CD is not yet completely understood. Autoimmunity is one factor that can partake in the triggering or modulation of inflammatory processes in IBD. The major zymogen-granule membrane glycoprotein 2 (GP2) has been recently identified as a major autoantigenic target in CD. Interestingly, GP2 is mainly expressed in the pancreas and has also been demonstrated to be a membrane-anchored receptor of microfold cells in the follicle-associated epithelium. Remarkably, GP2 is overexpressed at the site of CD inflammation in contrast to the one in UC. By utilizing novel enzyme-linked immunosorbent assays for the detection of GP2-specific IgA and IgG, the loss of tolerance to GP2 has been associated with a specific clinical phenotype in CD, in particular with the ileocolonic location of the disease.

## 1. Crohn's Disease 

The inflammatory bowel diseases (IBDs) are a group of inflammatory disorders of the colon and small intestine [1]. The ratio between females and males affected is around 1.35/1. The major clinical entities of IBD are Crohn's disease (CD) and ulcerative colitis (UC), demonstrating an increasing incidence of the former in developed countries with a North-South gradient decrease [1, 2]. Crohn's disease affects between 400,000 and 600,000 people in North America [3]. The estimated prevalence for Northern Europe ranges from 27 to 48 per 100,000 inhabitants [4]. Its annual incidence is approximately 3 cases per 100,000 in the United States and between 4 and 10 per 100,000 in Scandinavia and Germany. The disease occurs at any age, affecting a greater percentage of women over 20 years, but increasing numbers of cases are now being observed at younger age [5]. Smokers are two times more likely to develop Crohn's disease than nonsmokers [6].

The pathology in CD causes inflammation of all bowel wall layers, including the adventitia, and especially the terminal ileum, but can attack any part of the digestive tract, from the mouth to the anus [7]. The disease is manifested with tissue lesions, such as obstructions, fistulas and/or abscesses. Extended disease is associated with a higher incidence of carcinoma in particular in patients with Crohn's colitis.

The ileocolonic variant of Crohn's disease (CD), a disease of both the ileum and the large intestine, is the most common variant followed by Crohn's ileitis and Crohn's colitis. Using clinical diagnostic tools like endoscopy, the latter is rather difficult to differentiate from the colonic inflammation in ulcerative colitis (UC) in a number of cases [1].

Genetic factors, such as frameshift in the NOD2 gene (on chromosome 16 known as the CARD15 gene) [8, 9] or deletion of the XBP1 gene, have been identified as causative factors of CD [10]. To date, over thirty genes have been associated with CD. 

Due to its rising incidence in industrialized countries, it is also believed that also environmental component such as diet can be one of the causative factors of the disease [11]. Host-microbe interaction investigations have revealed a correlation between the responses to mycobacterial infections and host susceptibility to IBD [12]. 

Interestingly, autoimmunity is assumed to be another major contributor to the pathogenesis in IBD. Loss of tolerance to exocrine pancreatic, neutrophilic and intestinal goblet cellular targets is detected in CD and UC, respectively [14, 15]. Indirect immunofluorescence (IIF) was employed to establish pancreatic autoantibodies (PAB) as a specific marker of CD [15]. Pancreatic autoantibodies can be detected in up to 30% of patients with CD and in 68% of CD cases with extraintestinal complications such as idiopathic chronic pancreatitis [15–17]. The pathogenesis of CD remains elusive. The growing number of the patients and socioeconomic impact of the disease have created the demand for new diagnostic and therapeutic strategies. Thus, the recent discovery of pancreatic zymogen membrane glycoprotein 2 (GP2) as an autoantigenic target of CD-specific PAB can provide the opportunity to support this endeavor [18].

## 2. Glycoprotein 2 as a Gastrointestinal Autoantigen

Pancreatic secretory granule membrane GP2 is a 78  kDa glycoprotein, synthesized in the exocrine part of the pancreas by the acinus cells [19]. The heavily glycosylated GP2 carries carbohydrates at the N-terminus of the molecule and is linked to the zymogen-granule membrane through a C-terminal glycosyl phosphoinositol (GPI) anchor. Glycoprotein 2 represents the most abundant membrane protein of the acinus cells accounting for up to 40% of all granular membrane proteins [20, 21]. The acinus cells release GP2 together with the zymogens into the pancreatic ducts and ultimately into the intestine as a result of neuronal or hormonal stimulation of the pancreas. GP2 undergoes posttranslational modifications both in the cytoplasm of the acinus cells and subsequently after the cleavage of GPI anchor during secretion [22]. After cleavage from the membrane surface, GP2 as a self-binding glycoprotein forms soluble high-molecular aggregates in the pancreatic juice. Altogether, these GP2 variants expressing presumably different conformational epitopes may explain the two types of PAB patterns observed in IIF on pancreatic tissue.

In addition to the pancreatic location of GP2, this glycoprotein was also detected on the microfold cells (M cells) of intestinal Peyer's patches (PP) representing the follicle-associated epithelium (FAE) in the intestine [23]. Supporting these data, GP2 was also observed in lipid rafts of the brush-border membrane of enterocytes of rats in the small intestine [24]. The physiological function of GP2 is not yet completely known. However, GP2 seems to exert an immunomodulating and antimicrobial function in the intestine, as it has been reported for its homolog, the Tamm-Horsfall protein (THP) or uromodulin in the urogenital system [25, 26]. The most abundant urinary protein, THP, is secreted by renal tubular epithelial cells of the ascending limb of the loop of Henle. Much like GP2, THP bears a GPI anchor and a zona pellucida-like domain [27]. Another molecule with a zona pellucida-like domain expressed in ovarian and pancreatic tissue, the CUB/zona pellucida-like domain-containing protein (CUDZ1) has been also described to be reactive with PAB [28]. Thus, a cross-reactivity of PAB between these molecules cannot be excluded [29, 30].

## 3. The Physiological and Pathophysiological Functions of GP2 

Despite numerous studies to understand GP2's physiological function, the role of this glycoprotein needs to be further defined. Since GP2 was found to be expressed abundantly in the exocrine pancreas, an involvement in the regulation of granule formation had been proposed [31]. This hypothesis could not be corroborated in experiments using a GP2-deficient mice model, where the lack of GP2 did not influence zymogen-granule formation and secretion [32].

 Glycoprotein 2 and THP are derived from the duplication of a shared glycoprotein gene ancestor [33]. It is possible that these proteins could maintain similar functions but in different organ systems. It has been shown that THP is involved in antimicrobial defense, binding uropathogenic type 1 fimbriated *Escherichia coli* and preventing its interaction with urothelial (uroplakin) receptors [34]. Consequently, mice with impaired THP expression have an increased susceptibility to urinary tract infections [35].

An antimicrobial function can also be demonstrated for GP2, given that this protein can interact with type I pili (FimH) of specific enterobacteria, such as *Escherichia coli* and *Salmonella typhimurium* [36]. Moreover, GP2 can initiate mucosal immune responses by mediating the uptake of bacteria through intestinal M cells [23]. It has been shown that THP is a regulatory factor of innate and adaptive immunity in the urinary tract [26]. Similarly, GP2 could have the same role in the intestine as supported by several recent studies [13, 25, 37].

Thus, GP2 is upregulated on activated human T-cells and can be influenced by TNF*α* inhibition. Furthermore, GP2 decreases proliferation, apoptosis, and activation of human intestinal epithelial, mucosal, and peripheral T-cell; moreover it can modulate cytokine secretion, decreasing CXCL8 and upregulating TGF*β*1 [25]. These results seem to indicate that GP2 has an immunosuppressive and anti-inflammatory role. Due to the antimicrobial and immunomodulatory role, GP2 appears to be an essential factor maintaining the balance between tolerance to commensal bacteria and immune response against pathogens. The mucosal inflammation in CD may have its origin in the alteration of this balance, and, interestingly, the onset of inflammation in CD is supposed to be confined to the FAE, the side of GP2 expression in the intestine [38].

Altogether, these data suggest that GP2 has an important immunomodulating function in the intestine that is altered in CD patients. Given the plethora of new data on the physiological function of GP2, further efforts are required to understand the role of GP2 in the pathophysiology of CD.

## 4. Anti-GP2 Autoantibodies in CD and Association with the Clinical Phenotype

The assessment of anti-GP2 autoantibodies, in particular in combination with other IBD-specific markers such as the already established antibodies to *Saccharomyces cerevisiae* (ASCA) [39, 40], can be a powerful serological tool to discriminate the two main clinical entities of IBD, CD, and UC [41]. Novel enzyme-linked immunosorbent assays (ELISAs) for the routine testing of anti-GP2 autoantibodies have been developed recently, employing human recombinant GP2 as solid-phase antigen [42–44]. Combined determination of GP2 and CUZD1-specific autoantibodies by IIF using recombinantly expressed human embryonic-kidney cell autoantigens seems to have similar performance characteristics such as sensitivity and specificity [28, 45]. Thus, anti-GP2 autoantibodies are detected in 25–30% of patients with CD and in 5–12% of patients with UC [13, 41].

In a recent study, anti-GP2 and anti-ASCA IgA/IgG were significantly more abundant in CD patients with ileocolonic versus colonic location of the disease [46]. Altogether, anti-GP2 IgG and IgA appear to be associated with distinct disease phenotypes identifying patients at a younger age, with ileocolonic location, and stricturing behaviour with perianal disease [46]. The ileocolonic association was confirmed by another study evaluating the role of ileal inflammation in CD in the induction of humoral tolerance loss to GP2 [47]. However, Ob de Beeck et al. did not find an association with the clinical phenotype in CD using the same ELISA [44].

Our group reported the higher expression of GP2 in CD compared to UC in the intestine [18]. The FAE in the form of the PP is most abundant in the terminal ileum, and the frequency thereof corresponds to the rising number of microbiota in the distal small intestine [48]. Given the assumed onset of CD inflammation in the PP and the expression of GP2 as a specific antimicrobial receptor on M cells thereof, the frequency of anti-GP2 autoantibodies should be higher in patients with ileal involvement. 

This assumption is supported by the finding that acute gastroenteritis appears to be a triggering factor for the development of IBD, and a high prevalence of adherent-invasive *Escherichia coli *is a typical characteristic of CD patients [49, 50]. Although specific pathogenic species have not been yet strongly associated with CD, infectious agents recognized by gastrointestinal receptors may be involved in the induction of immune responses leading to disease and autoimmunity. This impaired tolerance towards a self-receptor molecule may be the onset of a strong humoral and cellular autoaggression, such as reported for the asialoglycoprotein receptor and its role in autoimmune liver diseases as the only liver-specific autoantigenic target [51, 52].

**Figure 1:**
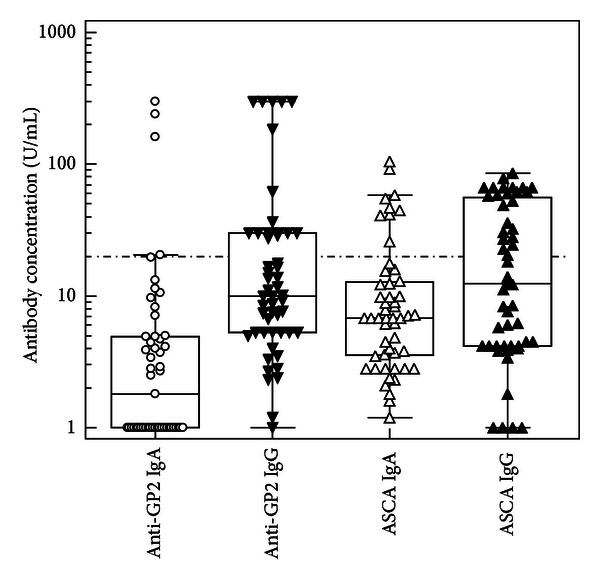
Comparison of anti-GP2 as well as ASCA IgA and IgG in 51 Italian patients with CD. The cutoff value of 20 arbitrary units (U/mL) recommended by the manufacturer of the commercial ELISAs (GA Generic Assays, Dahlewitz/Berlin, Germany) is indicated by a dotted line.

Further novel data regarding the association of antibodies to GP2 with location of disease have been reported recently. In a study presented as a poster (no. 164) at the 41st AMCLI Congress in Rimini in 2012, 83 Italian pediatric and adult patients with IBD were analyzed for the loss of tolerance to GP2 and ASCA reactivity. In fact, the detection of anti-GP2 autoantibodies by ELISA has also been found to be associated with ileocolonic location of the disease in the 39 patients with established location of CD (Table 1). Furthermore, autoreactivity to GP2 was determined in 9/15 (60.0%) of CD patients with stricturing behaviour and in 10/11 (90.9%) with moderate to severe endoscopic activity. In all 51 patients with CD, 58.8% (30/51) of the sera were positive for anti-GP2 antibodies and/or ASCA compared to 12.5% (4/32) of samples from patients with UC (*P* < 0.0001) (Figure 1). Healthy donors (*n* = 25) demonstrated no positivity for ASCA as well as anti-GP2 antibodies in this study. 

In another unpublished study, reporting novel data regarding the association of anti-GP2 antibodies with location of disease, 150 Jordanian patients (80 females and 70 males) with CD were analyzed for anti-GP2 antibody positivity. Out of the 150 patients, only 5 patients demonstrated stricturing or fistulizing disease behaviour. Remarkably, 4/5 patients revealed positive anti-GP2 IgA and/or IgG levels, whereas 39/145 of the remaining patients had elevated anti-GP2 levels (*P* = 0.0239). With regard to location of disease, 113 patients demonstrated ileal inflammation and 7 colonic location of disease, and in 30 patients both small and large intestines were affected. All 7 patients with colonic disease had no detectable anti-GP2 antibodies (0/7 versus 43/143, *P* = 0.1928). Out of 30 patients with the involvement of both parts of the intestine, 15 patients revealed elevated anti-GP2 antibody levels (15/30 versus 28/120, *P* = 0.0027). 

Altogether, these data seem to support the assumption that the loss of tolerance towards GP2 in CD patients is associated with severe disease behaviour and intestinal location of disease. Further studies with higher numbers of patients are needed to corroborate these findings.

**Figure 2:**
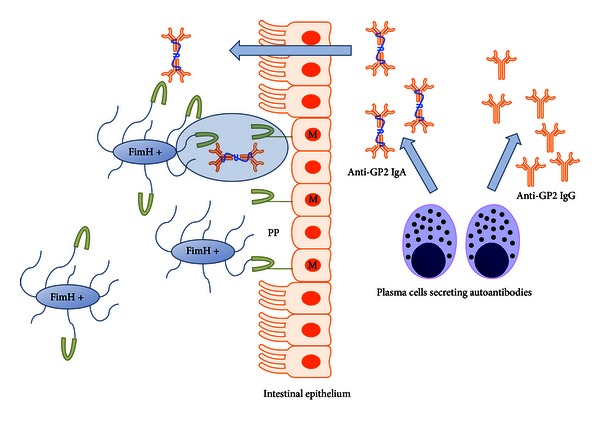
Glycoprotein 2 ((GP2) green) is synthesized by acinar pancreatic cells and released together with digestive enzymes into the intestinal lumen. Provided that GP2 is not digested by activated enzymes, GP2 can opsonise FimH positive microbes (FimH +) in the intestine. Additionally, GP2 has been demonstrated on the surface of microfold cells (M cells) of the Peyer's patches (PP) of the follicle-associated epithelium. This form of GP2 can interact with FimH positive bacteria and partake in the transcytosis thereof by the M cells. After the loss of tolerance towards GP2, plasma cells can synthesize anti-GP2 IgA which will be actively transported by the epithelium into the lumen of the intestine. The secreted anti-GP2 IgA can bridge FimH positive bacteria opsonised by pancreatic GP2 with the membrane-bound GP2 on the M cells (highlighted). Ultimately, this could lead to an overload of microbes in the mucosa due to an elevated transcytosis rate accelerating intestinal inflammation [13].

Given the rising number of studies after the recent discovery of autoantigenic targets in CD, it is tempting to speculate about a pathophysiological role of anti-GP2 autoantibodies or GP2 itself in the exacerbation or perpetuation of CD inflammation [13]. Thus, anti-GP2 IgG may diminish the suppressive effect of GP2 itself on T-cell activation and proliferation facilitating the onset or increasing the activity of the intestinal inflammation. Furthermore, anti-GP2 IgA, secreted as a dimeric form by intestinal epithelia into the gut lumen, can bridge GP2 of pancreatic origin bound to microbiota or pathogenic agents with GP2 on the surface of M cells facilitating the uptake thereof (Figure 2) [13]. The resulting increased transcytosis of GP2 opsonized pathogens can support the perpetuation of the inflammatory process in CD. 

## 5. Conclusions

Glycoprotein 2 is a novel autoantigenic target in Crohn's disease located mainly in the pancreas and follicle-associated epithelium of the intestine. 

Anti-GP2 antibodies, constituting novel CD-specific markers, are found to be positive in about 30% of patients with CD. 

Anti-GP2 IgG and IgA appear to be associated with distinct disease phenotypes.

Loss of tolerance towards GP2 is characteristic for patients with ileocolonic location of CD.

## Figures and Tables

**Table 1:** Association of anti-GP2 IgA and/or IgG with location of disease in CD.

*P* = 0.002	Anti-GP2 IgA/IgG		
	Positive	Negative	Total
Ileocolonic location			
Positive	11	5	16
Negative	4	19	23

Total	15	24	39

Investigating the association of anti-GP2 IgA/IgG with different clinical phenotypes in CD, 11/16 of CD patients with ileocolonic location demonstrated anti-GP2 antibody positivity (*P* = 0.002).

## Abbreviations

ASCA: Antibodies to *Saccharomyces cerevisiae *
CD: Crohn's disease
CUZD1: CUB/zona pellucida-like domain-containing protein
ELISA: Enzyme-linked immunosorbent assay
FAE: Follicle-associated epithelium
GP2: Zymogen-granule membrane glycoprotein 2
GPI: Glycosyl phosphatidylinositol
IBD: Inflammatory bowel disease
IIF: Indirect immunofluorescence
M cell: Microfold cell
PAB: Pancreatic autoantibodies
PP: Peyer's patches
THP: Tamm-Horsfall protein
UC: Ulcerative colitis.

## References

[1] Logan I, Bowlus CL (2010). The geoepidemiology of autoimmune intestinal diseases. *Autoimmunity Reviews*.

[2] Shivananda S, Lennard-Jones J, Logan R (1996). Incidence of inflammatory bowel disease across Europe: is there a difference between north and south? Results of the European collaborative study on inflammatory bowel disease (EC-IBD). *Gut*.

[3] Loftus EV, Schoenfeld P, Sandborn WJ (2002). The epidemiology and natural history of Crohn’s disease in population-based patient cohorts from North America: a systematic review. *Alimentary Pharmacology and Therapeutics*.

[4] Bernstein CN, Wajda A, Svenson LW (2006). The epidemiology of inflammatory bowel disease in Canada: a population-based study. *American Journal of Gastroenterology*.

[5] Baumgart DC, Sandborn WJ (2007). Inflammatory bowel disease: clinical aspects and established and evolving therapies. *The Lancet*.

[6] Cosnes J, Bourrier A, Nion-Larmurier I, Sokol H, Beaugerie L, Seksik P (2012). Factors affecting outcomes in Crohn's disease over 15 years. *Gut*.

[7] Lennard-Jones JE (1989). Classification of inflammatory bowel disease. *Scandinavian Journal of Gastroenterology, Supplement*.

[8] Ogura Y, Bonen DK, Inohara N (2001). A frameshift mutation in NOD2 associated with susceptibility to Crohn’s disease. *Nature*.

[9] Cuthbert AP, Fisher SA, Mirza MM (2002). The contribution of NOD2 gene mutations to the risk and site of disease in inflammatory bowel disease. *Gastroenterology*.

[10] Clevers H (2009). Inflammatory bowel disease, stress, and the endoplasmic reticulum. *The New England Journal of Medicine*.

[11] Baumgart DC, Carding SR (2007). Inflammatory bowel disease: cause and immunobiology. *The Lancet*.

[12] Jostins L, Ripke S, Weersma RK (2012). Host-microbe interactions have shaped the genetic architecture of inflammatory bowel disease. *Nature*.

[13] Roggenbuck D, Reinhold D, Werner L, Schierack P, Bogdanos DP, Conrad K (2013). Glycoprotein 2 antibodies in Crohn's disease. *Advances in Clinical Chemistry*.

[14] Saxon A, Shanahan F, Landers C, Ganz T, Targan S (1990). A distinct subset of antineutrophil cytoplasmic antibodies is associated with inflammatory bowel disease. *Journal of Allergy and Clinical Immunology*.

[15] Stöcker W, Otte M, Ulrich S, Normann D, Stöcker K, Jantschek G (1984). Autoantiköper gegen exokrines Pankreas und gegen intestinale Becherzellen in der Diagnostik des Morbus Crohn und der Colitis ulcerosa. *Deutsche Medizinische Wochenschrift*.

[16] Barthet M, Hastier P, Bernard JP, Bordes G, Frederick J, Allio S (1999). Chronic pancreatitis and inflammatory bowel disease: true or coincidental association?. *American Journal of Gastroenterology*.

[17] Conrad K, Schmechta H, Klafki A (2002). Serological differentiation of inflammatory bowel diseases. *European Journal of Gastroenterology and Hepatology*.

[18] Roggenbuck D, Hausdorf G, Martinez-Gamboa L (2009). Identification of GP2, the major zymogen granule membrane glycoprotein, as the autoantigen of pancreatic antibodies in Crohn’s disease. *Gut*.

[19] Hoops TC, Ivanov I, Cui Z, Colomer-Gould V, Rindler MJ (1993). Incorporation of the pancreatic membrane protein GP-2 into secretory granules in exocrine but not endocrine cells. *Journal of Biological Chemistry*.

[20] Ronzio RA, Kronquist KE, Lewis DS (1978). Glycoprotein synthesis in the adult rat pancreas. IV. Subcellular distribution of membrane glycoproteins. *Biochimica et Biophysica Acta*.

[21] Gmez-Lzaro M, Rinn C, Aroso M, Amado F, Schrader M (2010). Proteomic analysis of zymogen granules. *Expert Review of Proteomics*.

[22] Rindler MJ, Hoops TC (1990). The pancreatic membrane protein GP-2 localizes specifically to secretory granules and is shed into the pancreatic juice as a protein aggregate. *European Journal of Cell Biology*.

[23] Hase K, Kawano K, Nochi T (2009). Uptake through glycoprotein 2 of FimH + bacteria by M cells initiates mucosal immune response. *Nature*.

[24] Nguyen HTT, Amine AB, Lafitte D (2006). Proteomic characterization of lipid rafts markers from the rat intestinal brush border. *Biochemical and Biophysical Research Communications*.

[25] Werner L, Paclik D, Fritz C, Reinhold D, Roggenbuck D, Sturm A (2012). Identification of pancreatic glycoprotein 2 as an endogenous immunomodulator of innate and adaptive immune responses. *The Journal of Immunology*.

[26] Säemann MD, Weichhart T, Zeyda M (2005). Tamm-Horsfall glycoprotein links innate immune cell activation with adaptive immunity via a Toll-like receptor-4-dependent mechanism. *Journal of Clinical Investigation*.

[27] Fukuoka S, Freedman SD, Yu H, Sukhatme VP, Scheele GA (1992). GP-2/THP gene family encodes self-binding glycosylphosphatidylinositol-anchored proteins in apical secretory compartments of pancreas and kidney. *Proceedings of the National Academy of Sciences of the United States of America*.

[28] Komorowski L, Teegen B, Probst C (2012). Autoantibodies against exocrine pancreas in Crohn's disease are directed against two antigens: the glycoproteins CUZD1 and GP2. *Journal of Crohn's & Colitis*.

[29] Roggenbuck D, Humbel RL, Reinhold D, Bogdanos DP, Conrad K, Laass MW (2012). Glycoprotein 2 antibodies in inflammatory bowel disease: no association with disease phenotype?. *Journal of Pediatric Gastroenterology and Nutrition*.

[30] Roggenbuck D, Bogdanos D, Conrad K (2013). Loss of tolerance to one or two major targets in Crohn's disease or just cross-reactivity?. *Journal of Crohn's & Colitis*.

[31] Colomer V, Lal K, Hoops TC, Rindler MJ (1994). Exocrine granule specific packaging signals are present in the polypeptide moiety of the pancreatic granule membrane protein GP2 and in amylase: implications for protein targeting to secretory granules. *The EMBO Journal*.

[32] Yu S, Michie SA, Lowe AW (2004). Absence of the major zymogen granule membrane protein, GP2, does not affect pancreatic morphology or secretion. *Journal of Biological Chemistry*.

[33] Kobayashi K, Yanagihara K, Ishiguro K, Fukuoka S (2004). GP2/THP gene family of self-binding, GPI-anchored proteins forms a cluster at chromosome 7F1 region in mouse genome. *Biochemical and Biophysical Research Communications*.

[34] Pak J, Pu Y, Zhang ZT, Hasty DL, Wu XR (2001). Tamm-Horsfall protein binds to type 1 fimbriated Escherichia coli and prevents *E. coli* from binding to uroplakin Ia and Ib receptors. *Journal of Biological Chemistry*.

[35] Dou W, Thompson-Jaeger S, Laulederkind SJF (2005). Defective expression of Tamm-Horsfall protein/uromodulin in COX-2-deficient mice increases their susceptibility to urinary tract infections. *American Journal of Physiology*.

[36] Yu S, Lowe AW (2009). The pancreatic zymogen granule membrane protein, GP2, binds Escherichia coli type 1 Fimbriae. *BMC Gastroenterology*.

[37] Hölzl MA, Hofer J, Kovarik JJ (2011). The zymogen granule protein 2 (GP2) binds to scavenger receptor expressed on endothelial cells I (SREC-I). *Cellular Immunology*.

[38] Gullberg E, Söderholm JD (2006). Peyer’s patches and M cells as potential sites of the inflammatory onset in Crohn’s disease. *Annals of the New York Academy of Sciences*.

[39] Lakatos PL, Papp M, Rieder F (2011). Serologic antiglycan antibodies in inflammatory bowel disease. *American Journal of Gastroenterology*.

[40] Dotan I (2010). New serologic markers for inflammatory bowel disease diagnosis. *Digestive Diseases*.

[41] Bogdanos DP, Rigopoulou EI, Smyk DS (2011). Diagnostic value, clinical utility and pathogenic significance of reactivity to the molecular targets of Crohn's disease specific-pancreatic autoantibodies. *Autoimmunity Reviews*.

[42] Roggenbuck D, Reinhold D, Wex T (2011). Autoantibodies to GP2, the major zymogen granule membrane glycoprotein, are new markers in Crohn’s disease. *Clinica Chimica Acta*.

[43] Pavlidis P, Mytilinaiou MG, Roggenbuck D, Conrad K, Forbes A, Bogdanos DP (2011). Pancreatic GP2-specific autoantibodies are markers of Crohn's disease. *Gut*.

[44] Op de Beeck K, Vermeire S, Rutgeerts P, Bossuyt X (2010). Antibodies to GP2, the major zymogen granule membrane glycoprotein, in inflammatory bowel diseases. *Gut*.

[45] Kovacs M, Lakatos PL, Papp M (2012). Pancreatic autoantibodies and autoantibodies against goblet cells in pediatric patients with inflammatory bowel disease (IBD). *Journal of Pediatric Gastroenterology and Nutrition*.

[46] Bogdanos DP, Roggenbuck D, Reinhold D (2012). Pancreatic-specific autoantibodies to glycoprotein 2 mirror disease location and behaviour in younger patients with Crohn’s disease. *BMC Gastroenterology*.

[47] Pavlidis P, Romanidou O, Roggenbuck D (2012). Ileal inflammation may trigger the development of GP2-specific pancreatic autoantibodies in patients with Crohn's disease. *Clinical and Developmental Immunology*.

[48] Van Kruiningen HJ, West AB, Freda BJ, Holmes KA (2002). Distribution of Peyer’s patches in the distal ileum. *Inflammatory Bowel Diseases*.

[49] Darfeuille-Michaud A, Boudeau J, Bulois P (2004). High prevalence of adherent-invasive Escherichia coli associated with ileal mucosa in Crohn’s disease. *Gastroenterology*.

[50] Rodríguez LAG, Ruigómez A, Panés J (2006). Acute gastroenteritis is followed by an increased risk of inflammatory bowel disease. *Gastroenterology*.

[51] Rigopoulou EI, Roggenbuck D, Smyk DS (2012). Asialoglycoprotein receptor (ASGPR) as target autoantigen in liver autoimmunity: lost and found. *Autoimmunity Reviews*.

[52] Roggenbuck D, Mytilinaiou MG, Lapin SV, Reinhold D, Conrad K (2012). Asialoglycoprotein Receptor (ASGPR): APeculiar Target of Liver-Specific Autoimmunity. *Autoimmunity Highlights*.

